# Genome-wide analysis of the *PRT* gene family in rice reveals that *OsPRT7* plays a significant role in heat stress response

**DOI:** 10.1186/s12870-026-09070-z

**Published:** 2026-05-28

**Authors:** You Zhou, Chunni Wang, Manqiong Zhu, Wenhao Lv, FeiFan Ma, Chenghang Tang, Qiang Huang, XinTing Liu, Shuo Yang, Yingyao Shi

**Affiliations:** School of Agronomy, Bio-breeding Laboratory of Anhui Province, Anhui Agricultural University, 230036 Hefei, China

**Keywords:** Rice, Phosphoribosyl transferase, Heat stress, Haplotype, Gene family

## Abstract

PRTase plays vital roles in plant development and abiotic stress responses by maintaining the balance between plant growth and stress adaptation.The evolutionary conservation of the OsPRT gene family was elucidated by integrating phylogenetic tree construction, synteny assessments, and Ka/Ks ratio calculations. To identify prospective thermotolerant candidates, a comprehensive screening strategy was employed; this encompassed tissue-specific expression profiling, expression profiling under heat stress conditions, haplotype association analysis across 620 rice accessions, amino acid variant examination, and subsequent RT-qPCR verification. The promoter regions of *OsPRT* genes harbor multiple *cis*-acting elements related to stress responses. Notably, a single intra-species collinear paralogous pair was identified within the OsPRTs, with Ka/Ks evaluations strongly suggesting that this gene family has been subjected to rigorous purifying selection. Expression analysis indicated that most *OsPRT* genes have tissue-specific expression patterns. Haplotype analysis revealed significant differences among *OsPRT5*, *OsPRT6*, and *OsPRT7*. RT-qPCR results showed that the expression level of OsPRT7 exhibited a downward trend under high-temperature treatment. Collectively, our results suggest that *OsPRTs* have potential functions in rice heat stress tolerance, providing new insights into the molecular mechanisms underlying the environmental adaptation and adaptive evolution of *OsPRT* genes in rice.

**Supplementary Information:**

The online version contains supplementary material available at 10.1186/s12870-026-09070-z.

## Introduction

Global warming poses serious threats to agricultural production and global food security. It has been estimated that every 1 °C increase in global average temperature will cause 3%–8% declines in the yields of major staple crops worldwide [1]. Heat stress has great impacts on the seasonal growth and geographical distribution of crops [2].Thus, generating crop cultivars with enhanced thermotolerance is critical to securing continuous agricultural production, given that the global population is forecast to reach the 10-billion mark by 2050 [3–6]. Sustaining nearly half of the worldwide demographic, rice stands as a cornerstone of global agricultural production and human nutrition [7, 8]. However, rice production is adversely affected by heat stress associated with global warming [9, 10]. Heat stress significantly affects rice growth and development, particularly at the flowering stage. While high temperatures during the vegetative phase are detrimental, heat stress incurred at the reproductive or flowering stage is profoundly more destructive to rice physiology. This heightened vulnerability manifests as severe impairments in critical reproductive events, namely panicle formation, gametophyte maturation, anthesis, pollination dynamics, and subsequent fertilization [11–13]. An average temperature above 33 °C during meiosis in rice panicles will lead to failure in normal development of floral organs and pollens, thereby resulting in reduced seed-setting rate and abnormal floret development [14]. Grain filling is a critical stage for the formation of rice yield and quality. Heat stress during grain filling accelerates the grain-filling rate and shortens the grain-filling duration, which not only affects the grain weight but also impairs the rice quality [15].

Glycosylation plays a vital physiological role in all life processes in plants, including growth, development, and stress responses [16, 17]. Phosphoribosylation is a specialized glycosylation modification catalyzed by phosphoribosyltransferases (PRTases). It is a key step in the biosynthetic pathways of purine and pyrimidine nucleotides, tryptophan (Try), histidine (His), and the cofactor NAD(P), and plays important roles in regulating the production of these metabolites [18]. In plants the synthesis of purine nucleotides occurs through either *de novo* synthesis or salvage pathways via a complex sequence of enzymatic reactions. The crucial enzymes of the salvage pathway are adenine phosphoribosyltransferase (APRT) and adenosine kinase for the synthesis of AMP and hypoxanthine/guanine phosphoribosyltransferase (HGPRT) and hypoxanthine/guanine kinase for the synthesis of IMP and GMP [19, 20].From the perspective of molecular catalytic mechanism, the core function of PRTases is to catalyze the transfer of the ribose-5-phosphate group from the glycosyl donor phosphoribosyl pyrophosphate (PRPP) to acceptor molecules such as adenine, guanine and uracil, thereby promoting the formation of glycosidic bonds [21]. As a core member of the PRTase family, adenine phosphoribosyltransferase (APRT) possesses dual physiological functions. It not only acts as a key enzyme mediating the salvage synthesis of adenine bases and maintaining normal nucleotide biosynthesis, but also converts active cytokinin bases into inactive nucleotide forms, thereby participating in the regulation of plant hormone homeostasis [19]. In recent years, the functions of APRT family genes have been preliminarily characterized in several plants. Chun et al. revealed that heat-induced changes in the expression pattern of *OsAPT2* in young panicles may, at least partially, mediate thermo-sensitive genic male sterility (TGMS) in Annong S-1. Furthermore, they used an antisense strategy to silence the homologous gene of *OsAPT2* in *Arabidopsis thaliana*. The resultant homozygous transgenic plants displayed decreased AMP content and pollen germination rate, along with distinct abnormalities in leaf phenotype and flowering time [22]. CRISPR/Cas9 editing of two adenine phosphoribosyltransferase‑encoding genes in common bean (Phaseolus vulgaris L.) revealed that PvAPRT1 is primarily involved in adenine salvage, while PvAPRT5 acts as the predominant isoform in modulating cytokinin homeostasis and stress responses, and exerts crucial regulatory effects on root and nodule growth [23].However, research over the past few decades on plant PRTases has been mainly focused on their roles in plant survival and growth, while has largely ignored their functions and interactive effects in plant development and abiotic stress responses [24]. In fact, some recent studies have clearly shown that PRTase-related metabolic pathways are crucial for chloroplast development, gametophyte development, and salt and osmotic stress responses [25–27].

In this study, we identified the *PRT* gene family members and analyzed their expression patterns in rice under heat stress with bioinformatic approaches. We further screened and obtained a key candidate gene *OsPRT7* by combining haplotype analysis and gene expression profiling. The findings not only improve our understanding of the abiotic stress response mechanism in rice, but also provide important genetic resources and a theoretical foundation for subsequent genetic improvement of heat tolerance and molecular-assisted breeding in rice.

## Results

### Identification and phylogenetic analysis of the *OsPRT* family members

**Fig. 1:**
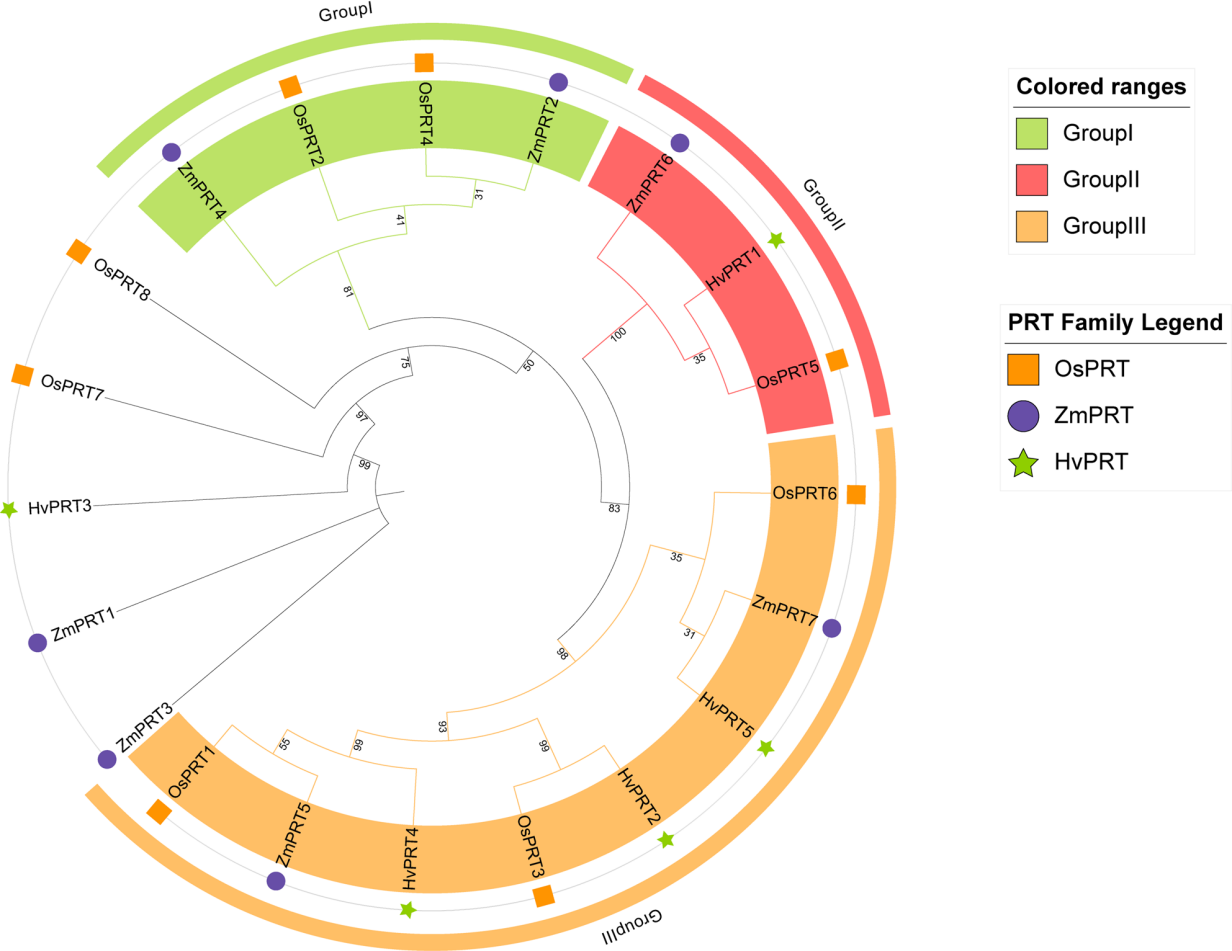
Phylogenetic analysis of *OsPRT*, *ZmPRT*, and *HvPRT* members. Different colors represent different subfamilies

We identified eight OsPRTs, seven ZmPRTs, and five HvPRTs for multiple sequence alignment and construction of a phylogenetic tree using TBtools (Fig. 1, Table S1). The *PRT* members were divided into three subgroups on the branches of the phylogenetic tree, where Group II had the fewest members (1 *ZmPRT* member, 1 *HvPRT* member, and 1 *OsPRT* member), whereas Group III had the most members (2 *ZmPRT* members, 3 *HvPRT* members, and 3 *OsPRT* members).

### Promoter *cis*-acting element analysis of *OsPRT* members

**Fig. 2:**
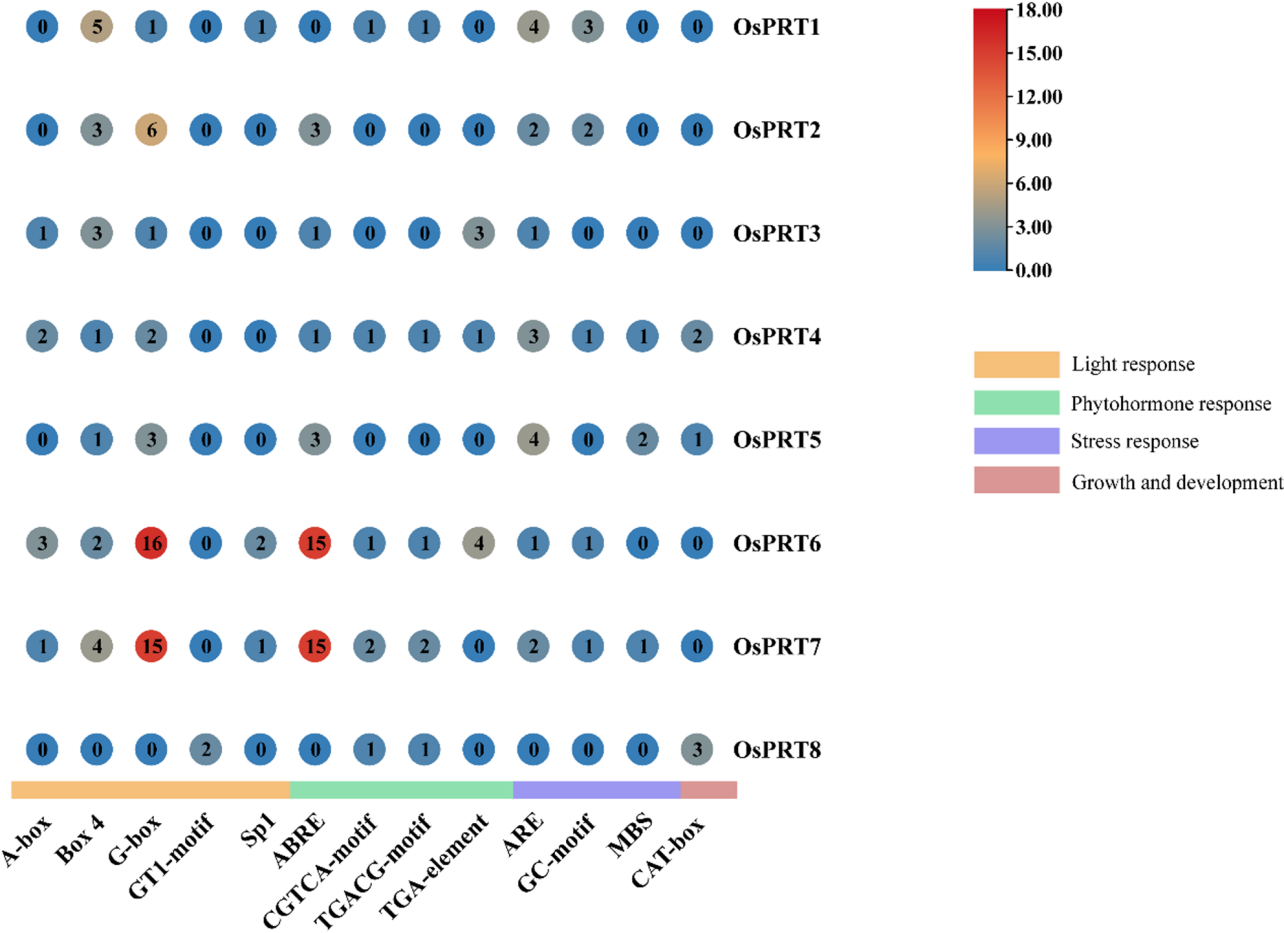
Analysis of *cis*-acting elements in *OsPRT* genes. Heatmap analysis of *cis*-acting elements in the promoter regions of *OsPRT* genes. In the heatmap, the values represent the numbers of different *cis*-acting elements, with a darker color indicating a larger number

PlantCARE was used to predict the *cis*-acting elements in the 2000-bp upstream sequences of *OsPRT* genes. As depicted in the quantitative distribution in Fig. 2, the G-box motif exhibited the highest frequency of occurrence among all identified light-responsive regulatory sequences (44 occurrences, accounting for 57.90% of the total). Among the plant hormone-responsive elements, ABA-responsive element (ABRE) was the most frequent element (38 occurrences, representing 65.51% of the total). For the stress-responsive elements, stress-responsive key element (ARE) was the most prevalent element (17 occurrences, accounting for 68% of the total). The over-representation of ABRE suggests that OsPRTs may function as downstream targets within the abscisic acid (ABA)-dependent signaling network. Furthermore, the abundance of ARE elements posits their potential involvement in maintaining oxidative homeostasis under thermal stress conditions. The coexistence of these dual regulatory modules likely empowers OsPRTs to synergistically integrate ABA-mediated signaling with thermal stress adaptation, thereby facilitating a coordinated defensive response in rice.

### Collinearity analysis of *OsPRTs*

**Fig. 3:**
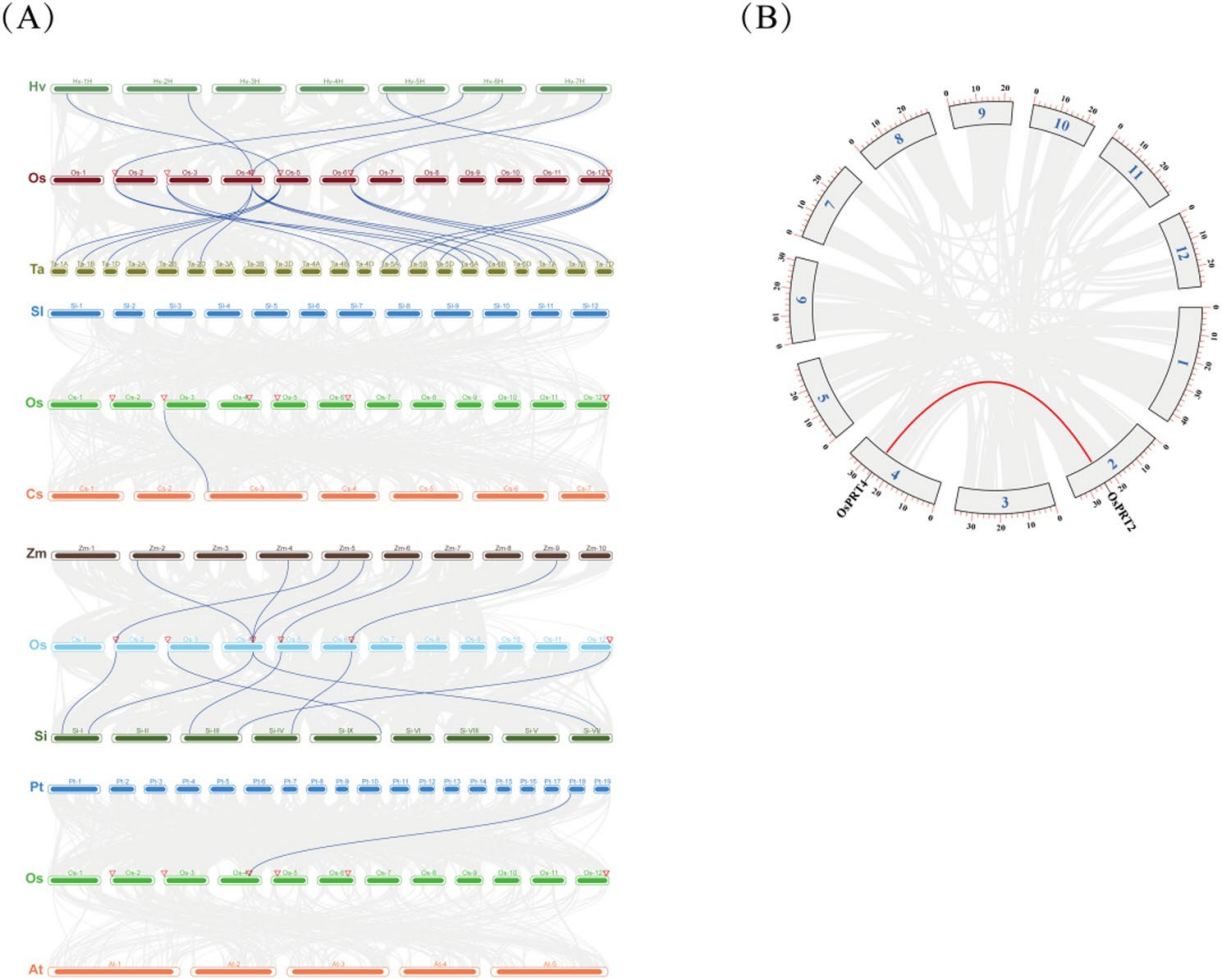
Collinearity analysis of *PRT* genes within rice and between Oryza sativa and Hordeum vulgare, Triticum aestivum, Setaria italica, Cucumis sativus, Solanum lycopersicu, Zea mays, *Populus trichocarpa*, and *Arabidopsis thaliana*. **A** Collinearity analysis of *PRT* genes among Oryza sativa, Hordeum vulgare, Triticum aestivum, Setaria italica, Cucumis sativus, Solanum lycopersicu, Zea mays, Populus trichocarpa, and Arabidopsis thaliana. **B** Intraspecific collinearity analysis of *PRT* genes in rice

To further elucidate the evolutionary relationships of the *PRT* gene family across different plant species, we performed a genome-wide collinearity analysis of *PRT* genes using MCScanX in different plant species including *Oryza sativa*, *Hordeum vulgare*, *Triticum aestivum*, *Setaria italica*, *Zea mays*, *Cucumis sativus*, *Solanum lycopersicum*, *Populus trichocarpa*, and *Arabidopsis thaliana* (Fig. 3). There were 6, 18, 6, and 6 collinear *PRT* gene pairs between *O. sativa* and *H. vulgare*, *T. aestivum*, *S. italica*, and *Z. mays* (monocotyledonous plants), while only 1, 0, 1, and 0 collinear *PRT* gene pairs between O. sativa and C. sativus, S. lycopersicum, *P. trichocarpa*, *A. thaliana* (dicotyledonous plants), respectively. These results indicated that the *PRT* gene family may be more conserved during the evolution of monocotyledonous plants. We then analyzed the duplication events of *OsPRTs* on different chromosomes in the rice genome using MCScanX, resulting in the identification of one pair of collinear genes (*OsPRT2*/*OsPRT4*) (Fig. 3). By estimating the ratio of non-synonymous to synonymous substitutions (Ka/Ks) across the homologous sequences, we further investigated the nature of the selective forces driving the evolution of the OsPRT gene family (Table 1). Evolutionary analysis revealed that the OsPRT2/OsPRT4 pair has been subjected to substantial purifying selection, as evidenced by a calculated Ka/Ks index of 0.175.

**Table 1:** Ka/Ks ratios of OsPRT homologous genes

Gene 1	Gene 2	Ka	Ks	Ka/Ks
OsPRT2	OsPRT4	0.107	0.611	0.175

### Tissue-specific expression analysis and expression analysis of *OsPRTs* under heat stress

**Fig. 4:**
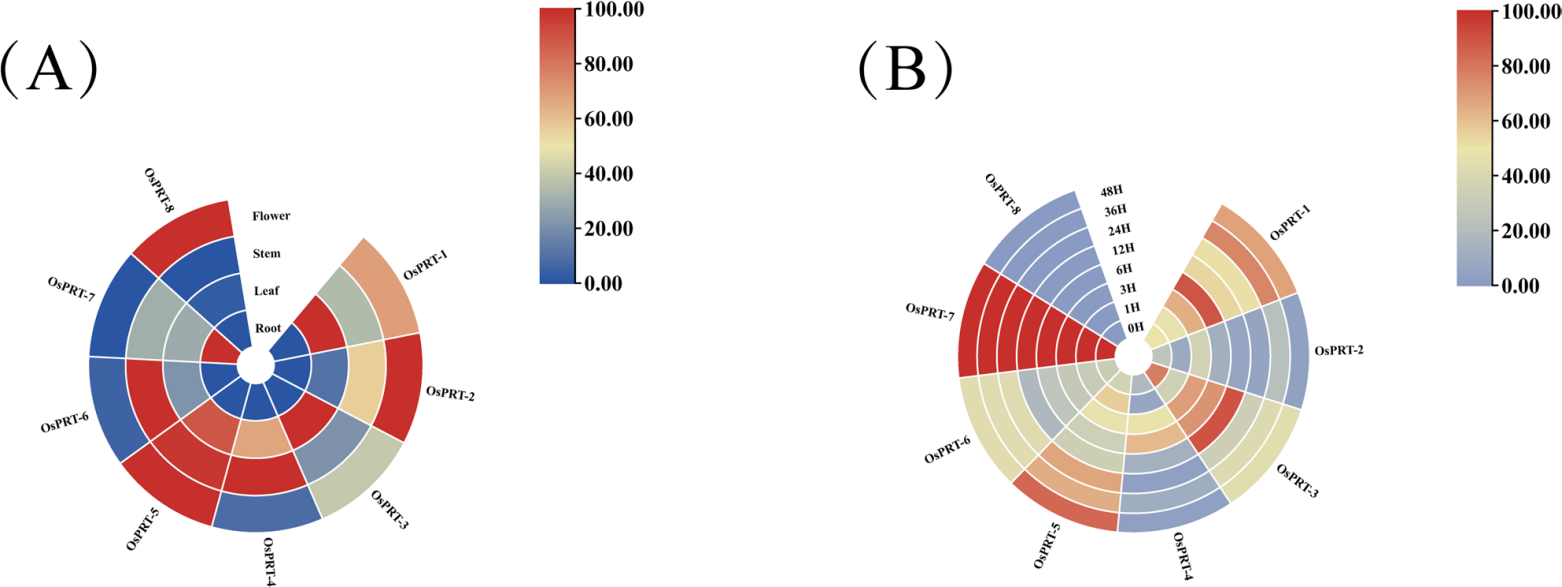
Expression analysis of *OsPRTs*. **A** Tissue-specific expression analysis of *OsPRTs*. **B** Expression analysis of *OsPRTs* under heat stress. The FPKM values represented by different colors in the figure are shown in the color bar on the right

The expression patterns of the OsPRT family across various rice tissues and in response to thermal stress were retrieved from the Rice RNA-seq Database. These data were subsequently rendered as heatmaps utilizing the TBtools software suite (Fig. 4). Members of the OsPRT family exhibited a pervasive expression profile across a broad spectrum of rice tissues. Substantial transcriptional divergence was evident among individual paralogs within any given anatomical context. Based on the tissue specificity index (TAU, 0.15–0.85) calculated from the FPKM values, a total of 7 OsPRT members with higher levels of expression were identified, including OsPRT1, OsPRT2, OsPRT3, OsPRT4, OsPRT5, OsPRT6, OsPRT7. The heat stress expression profiles revealed that, except for OsPRT8, the other seven OsPRT family members were induced by heat stress and exhibited dynamic expression patterns, which could serve as candidate genes for heat stress research in rice.

### Haplotype analysis of *OsPRTs*

**Fig. 5:**
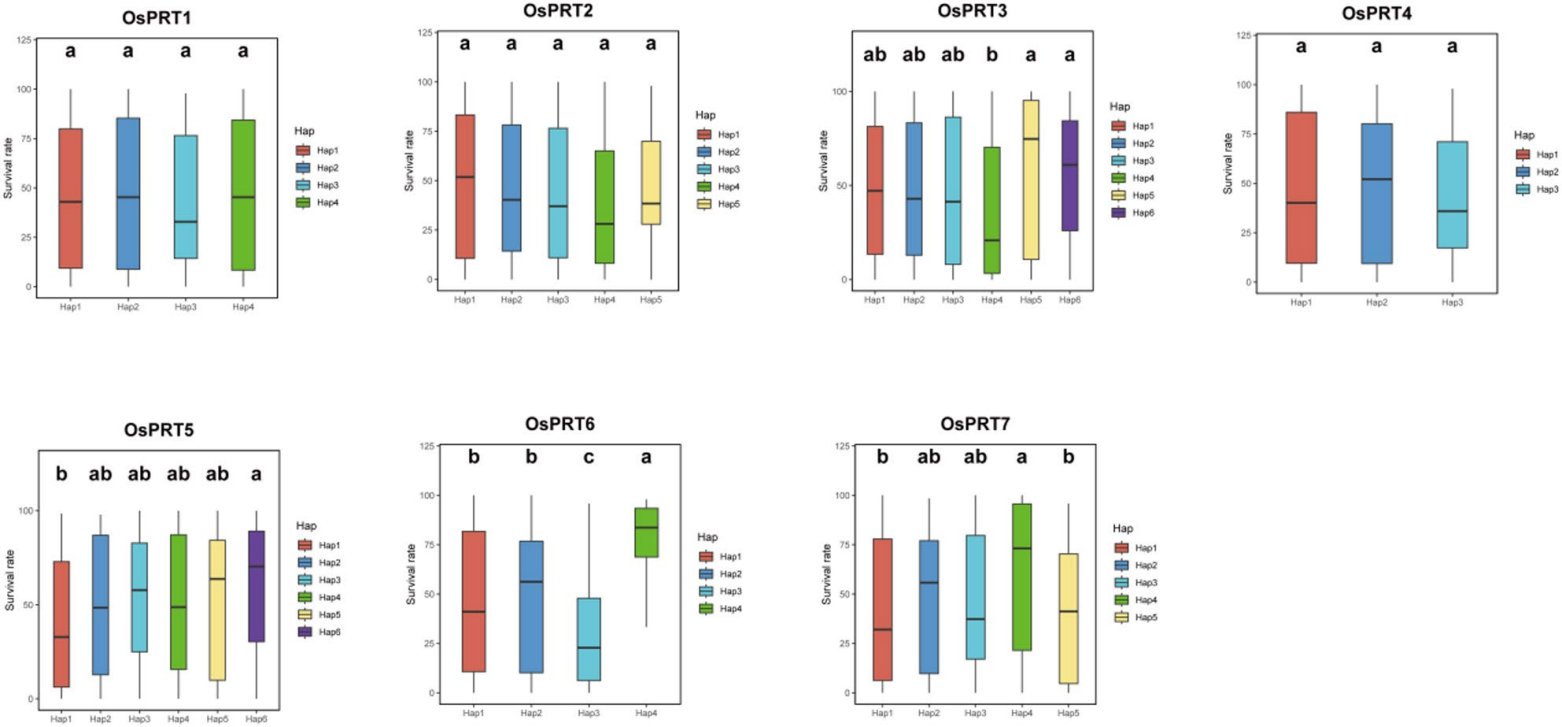
Haplotype analysis of seven *OsPRTs* with high expression. Different colors represent different haplotypes, and the letters in multiple comparison annotations indicate significant differences among haplotypes

To elucidate the involvement of OsPRT genes in rice thermotolerance, seven highly transcribed members of this family were subjected to comprehensive genotyping and phenotypic analyses (Fig. 5, Table S2). Among these genes, the haplotypes of only four genes (*OsPRT3*, *OsPRT5*, *OsPRT6*, and *OsPRT7*) were genotyped. Three out of these four genes exhibited significant differences (*p* < 0.05) (Table S3).

### Amino acid site mutation and secondary structure prediction of candidate *OsPRTs*

**Fig. 6:**
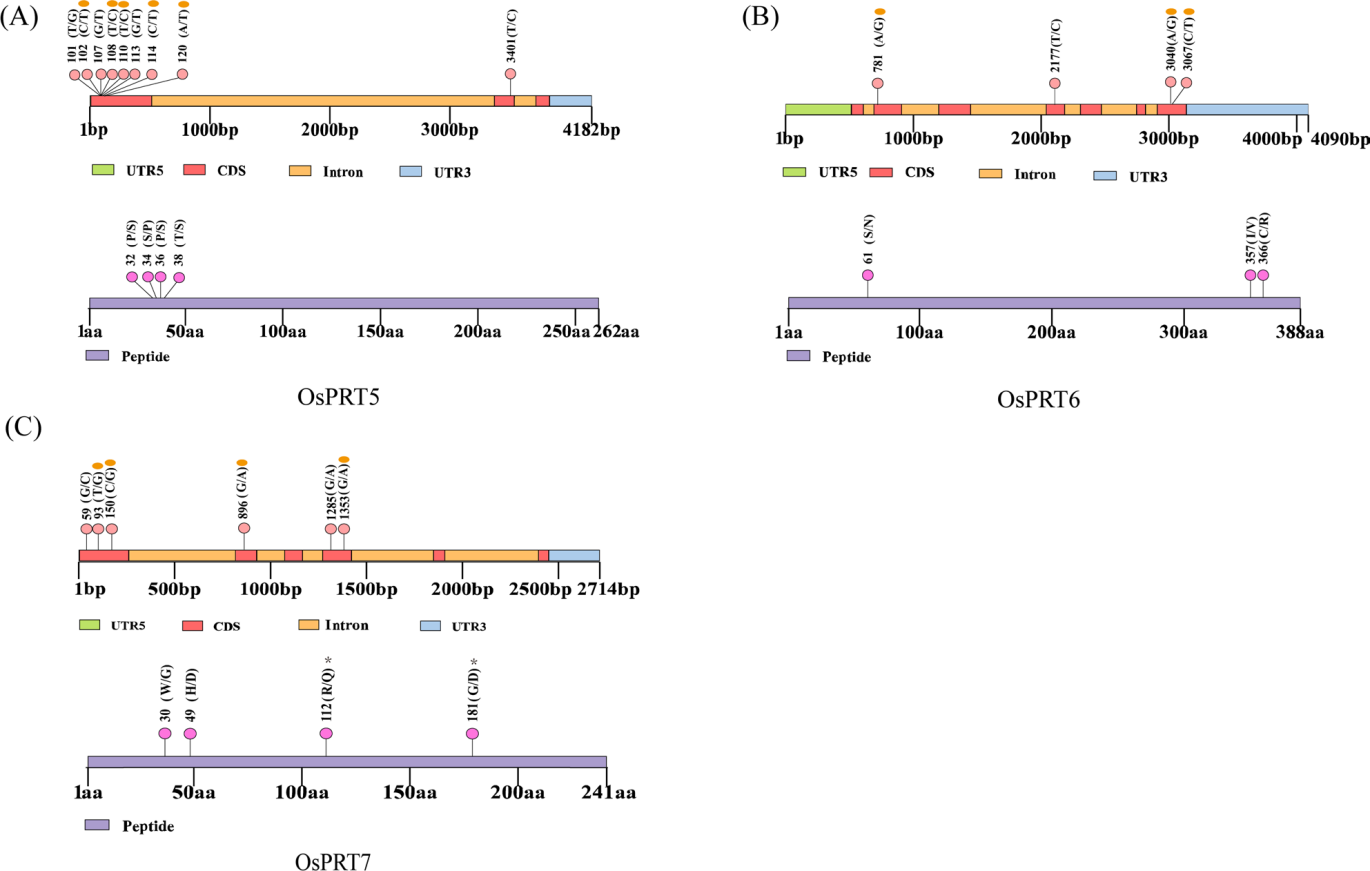
Mutation analysis of single-nucleotide polymorphism (SNP) sites in candidate *OsPRT* members. **A** SNP sites mutation in OsPRT5. **B** SNP sites mutation in OsPRT6. **C** SNP sites mutation in OsPRT7. The 5’ untranslated region (UTR5) sequences are represented by green squares, the coding DNA sequence (CDS) by red squares, the intron sequences by yellow squares, the 3’ untranslated region (UTR3) sequences by blue squares, and the amino-acid sequences by purple squares. The SNPs denoted by yellow ellipses are non-synonymous SNPs. The amino-acid substitutions indicated by five-pointed stars occur within the conserved motifs of the genes

To further substantiate the aforementioned candidate loci, the specific sequence variations within the haplotypes exhibiting significant differences were subjected to detailed characterization (Fig. 6). As a result, 9, 4, and 6 nucleotide mutations were identified in *OsPRT5*,* OsPRT6*, and *OsPRT7*, among which 4, 3, and 4 were non-synonymous mutations, respectively. Among these three genes, only one gene (*OsPRT7*) contained two amino acid substitutions located on conserved motifs (Figure S1). Deleterious mutation prediction indicated that the R112Q substitution in *OsPRT7* was deleterious (Table S4). In silico prediction using the SOPMA server revealed that the secondary structural composition of OsPRT7 is constituted by four primary elements: α-helix, β-sheet, β-turn, and random coil (Table S5). Notably, non-synonymous SNPs resulting in amino acid substitutions precipitated structural rearrangements, leading to shifts in the proportions of α-helix and β-sheet within these proteins. For instance, upon SNP induction, the proportion of α-helix in OsPRT7 rose from 30.42% to 32.92%, while that of β-sheet dropped from 15.00% to 13.75%. Therefore, based on the amino acid mutation, *OsPRT7* was further confirmed as a potential key candidate gene for heat-stress tolerance in rice.

### RT-qPCR of *OsPRTs*

**Fig. 7:**
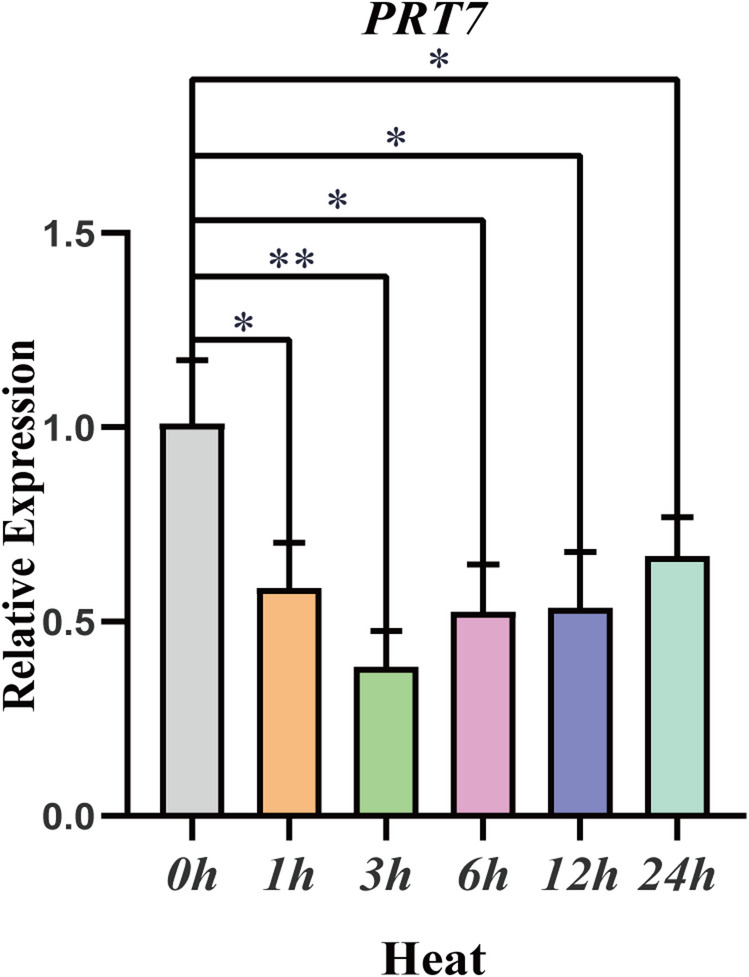
Expression analysis of the *OsPRT7* genes under heat treatment. Statistical analysis was performed using WPS 2023, and analysis of variance (ANOVA) was conducted with IBM SPSS Statistics 25. The significance levels were defined as *** *p* < 0.001, ** *p* < 0.01, * *p* < 0.05

The relative expression of *OsPRT7* under heat stress was analyzed by RT-qPCR (Fig. 7). After heat stress treatment, the expression level of *OsPRT7* was significantly downregulated and showed dynamic changes with increasing stress duration, suggesting that this gene may negatively regulate the heat stress response in rice and is a key candidate gene involved in heat tolerance in the *OsPRT* family.

## Discussion

Our results revealed that rice harbors eight *PRT* genes distributed across six chromosomes. Although rice has a smaller genome than *Z. mays* and *H. vulgare*, it has a relatively large number of *PRT* genes, which may be attributed to a higher frequency of gene duplication events. There seems to be no correlation between the genome size and number of the *PRT* gene family members, indicating that this gene family may have undergone substantial gene loss during evolution [28]. In *O. sativa*, only two *PRTs* were originated from whole-genome duplication or segmental duplication. Our Ka/Ks analysis also indicated that *PRT2* and *PRT4* are highly conserved. The phylogenetic analysis among *PRT* gene family members in *Z. mays*, *O. sativa*, and *H. vulgare* revealed that the *PRT* gene family members form three distinct evolutionary subgroups, encompassing multiple independent lineages. These results strongly suggest an evolutionary pattern for this gene family, where the core members have been conserved, while the peripheral members exhibit high degrees of evolutionary diversification.

Promoter regions play pivotal roles in plant gene expression in complex and diverse natural environments. The type and amount o*f cis*-acting elements in the promoter sequences can indicate the response of genes to inducing factors [29]. ABA has a variety of physiological functions, including increasing plant stress resistance, causing stomatal closure, promoting seed dormancy, and regulating seed embryo development [30, 31]. Our analysis of the promoter regions of the *OsPRT* gene family revealed that their promoter regions may be responsive to various external stimuli such as hormones and stresses. In particular, *OsPRT6* and *OsPRT7* contain as many as 15 ABRE elements, which is far greater than that of other *OsPRT* genes.

The pattern of substitutions at amino acid sites in proteins can strongly indicate their biophysical and functional importance and the selection pressures acting at individual sites [32]. For example, Ullah et al. reported that the amino acid residue Y346 plays a critical role in ion selectivity, and mutation at this site can significantly enhance tolerance to LiCl salt stress. Other mutants (Y346F, A399V, and Y346A) only showed slightly improved NaCl tolerance, suggesting that such mutations have the potential to enhance salt tolerance in intact plants [33]. Feng et al. found that among the five amino acid substitutions, mutation at residue 3 successfully reduced the lipid and TAG synthesis capacity of *HaDGAT2*, while enhanced the total lipid and TAG synthesis capacity of *ClDGAT2* [34]. Our study found that the candidate gene *OsPRT7* harbors SNP variations and amino acid substitutions, and some of the amino acid substitution sites are located within conserved motifs. In accordance with the findings of Zhu et al., the attenuation of mean particle dimensions is fundamentally underpinned by a conformational transition, characterized by a diminished proportion of β-sheets alongside an enrichment in .α-helix, β-turns, and disordered random coils [35]. Consequently, modifications in the secondary structural architecture may play a pivotal role in modulating the biochemical properties and overall functional integrity of these plant proteins. Notably, our results showed that SNP-induced amino acid variations resulted in a substantial increase in α-helix content and a decrease in β-sheet content in OsPRT7. These conformational transitions are capable of reconfiguring the stoichiometric balance and spatial arrangement of secondary structural motifs, thereby precipitating the functional divergence of the resulting OsPRT variants.

## Materials and methods

### Identification of the *OsPRT* members and phylogenetic analysis of the *PRT* gene family

The corresponding hidden Markov model (HMM) profile for the OsPRT family (accession number PF00156) was acquired directly from the Pfam database.To identify putative OsPRT family members, the complete rice proteome was scanned utilizing the HMMER 3.0 software suite, applying a stringent expectation value (E-value) cutoff of < 1e − 15 [36]. The Ensembl database served as the primary source for acquiring all necessary genomic DNA sequences and associated annotation files. Phylogenetic reconstruction was performed within the TBtools software suite employing the Maximum Likelihood (ML) algorithm. Specific evolutionary parameters included the LG substitution model, a four-category discrete gamma distribution, and a pairwise deletion treatment for gaps, with nodal support validated through 5000 bootstrap replicates. To ensure the reliability of the phylogenetic classification, only clades substantiated by a rigorous bootstrap threshold of 80% were recognized as well-supported subgroups.The categorization of individual gene subfamilies was inferred from the calculated evolutionary distances within the phylogenetic reconstruction. For enhanced visualization and structural annotation, the resulting phylogenetic tree was stylized using the iTOL online platform (https://itol.embl.de/) [37].

### *Cis*-acting elements and collinearity analysis

The 2000-bp fragment upstream of *OsPRTs* was used as the promoter sequence, and PlantCARE was employed to analyze the *cis*-acting elements in the promoter region [38]. MCScanX was used to analyze the collinearity of duplicated gene pairs among *OsPRT* family members and across eight different species (At, Sl, Cs, Cl, Hv, Ta, Zm, and Si) [39]. Visualization of the analytical data was performed through the TBtools software suite [40]. The frequencies of non-synonymous (Ka) and synonymous (Ks) substitutions were derived for each paralogous OsPRT pair using the KaKs_Calculator software [41].

### Tissue-specific expression and expression data of *OsPRTs* under heat stress

Spatiotemporal expression patterns of OsPRT genes across diverse tissues and developmental stages, alongside transcriptomic datasets under thermal stress, were retrieved from the Rice RNA-seq database [42]. The expression level data were standardized using GraphPad Prism, and a heatmap was generated using TBtools [40]. The TAU index (Tissue specificity index) of the FPKM values of the *OsPRTs* members in rice were calculated using the TAU Calc tool in TBtools, with a threshold range set between 0.15 and 0.85, to filter the tissue-specific levels of expression of the *OsPRT* genes [43].

### Heat stress treatment and qRT-PCR

Rice seeds (Nipponbare) were disinfected with 3% sodium hypochlorite for 30 min, germinated at 28 °C for three days, and then transplanted into hydroponic boxes filled with Hoagland nutrient solution. Rice seedlings were cultivated in a precision-controlled growth chamber under a consistent diurnal cycle (28 °C/28°C day/night). The environmental parameters were maintained at a relative humidity of 75% and a photosynthetic photon flux equivalent to 20,000 lx, with a 12-hour photoperiod. The seedlings were treated with high temperature (42 °C). Rice leaf samples were harvested at designated time intervals (0, 1, 3, 6, 18, and 24 h post-treatment), snap-frozen in liquid nitrogen, and subsequently preserved at − 80 °C to maintain RNA integrity for future extraction.To obtain a fine powder, the harvested tissues were pulverized under cryogenic conditions using a pre-chilled mortar and pestle in the presence of liquid nitrogen. Total RNA isolation was performed using the MiniBEST Plant RNA Extraction Kit according to the manufacturer’s instructions. First-strand cDNA synthesis was performed using a Reverse Transcription Kit, effectively converting the isolated RNA templates into stable cDNA. The relative transcript abundance of the target genes was quantified through quantitative real-time PCR (qRT-PCR) assays. Specific primer pairs targeting the OsPRT7 locus were tailored(Table S6). To ensure the accuracy of the quantification, the relative transcript levels were standardized against the endogenous control gene OsActin1 (LOC_Os03g61970) [44]. Fluorescence-based quantitative data were acquired utilizing the LightCycler 96 real-time PCR system (Roche, Switzerland) to monitor the amplification process. The qPCR reactions were executed in a total volume of 20 µL, comprising 10 µL of AceQ Universal SYBR qPCR Premix, 2 µL of cDNA template, and 0.8 µL of each specific primer, with the remaining volume adjusted to 20 µL using 6.4 µL of nuclease-free ddH2O. The thermal cycling profile initiated with an initial denaturation at 95 °C for 5 min, followed by 40 amplification cycles. Each cycle consisted of a 10-s denaturation at 95 °C and a combined annealing/extension step at 60 °C for 30 s. Sampling was conducted at six time points: 0, 1, 3, 6, 12, and 24 h, with three replicates for each time point. For each replicate, five rice seedlings were pooled for processing. In total, ninety rice seedlings were used for each treatment. Three biological replicates and three technical replicates were carried out. Relative fold-change in the transcript levels of each target gene was determined based on the 2^−ΔΔCt^ analytical method [45]. Primary data processing was conducted using WPS 2025, while GraphPad Prism 8 was utilized for comprehensive analysis of variance and the subsequent generation of high-resolution graphics.

### Haplotype analysis and amino acid site mutation of *OsPRTs*

The association between allelic variations and thermal sensitivity was investigated through haplotype analysis of the OsPRT family, utilizing a diversity panel of 620 natural rice germplasms with established heat tolerance phenotypes [46]. SnapGene (version 6.0.2) was used to analyze whether mutations occurred at the nucleotide and amino acid sites of *OsPRT* members. The spatial distribution of amino acid substitutions within the OsPRT protein family was mapped utilizing the lollipop plot functionality within the R environment. Identification of conserved structural motifs within the OsPRT protein sequences was executed utilizing the MEME discovery environment, with the search parameters configured to detect a maximum of 10 distinct motifs [47]. To evaluate the potential functional consequences of non-synonymous variations, the PROVEAN tool was employed to infer whether these amino acid substitutions exerted deleterious effects on protein function(accessed January 30th, 2026; ( http://provean.jcvi.org/seq_submit.php).

## Supplementary Information

Supplementary Material 1.

Supplementary Material 2.

## Data Availability

The datasets generated and analyzed during the current study are available in the Mendeley Data repository (https://data.mendeley.com/preview/3t73gj43wg?a=b24c8490-b51e-4363-bf44-7fc7f221128c). All data generated or analyzed during this study are included in this published article and its supplementary information files.
