# Supplementary figures and images for: Genome-wide analysis of the PRT gene family in rice reveals that OsPRT7 plays a significant role in heat stress response
Source: BMC Plant Biol. 2026 May 28;26:1261. doi: 10.1186/s12870-026-09070-z (PMC13403581; doi:10.1186/s12870-026-09070-z)

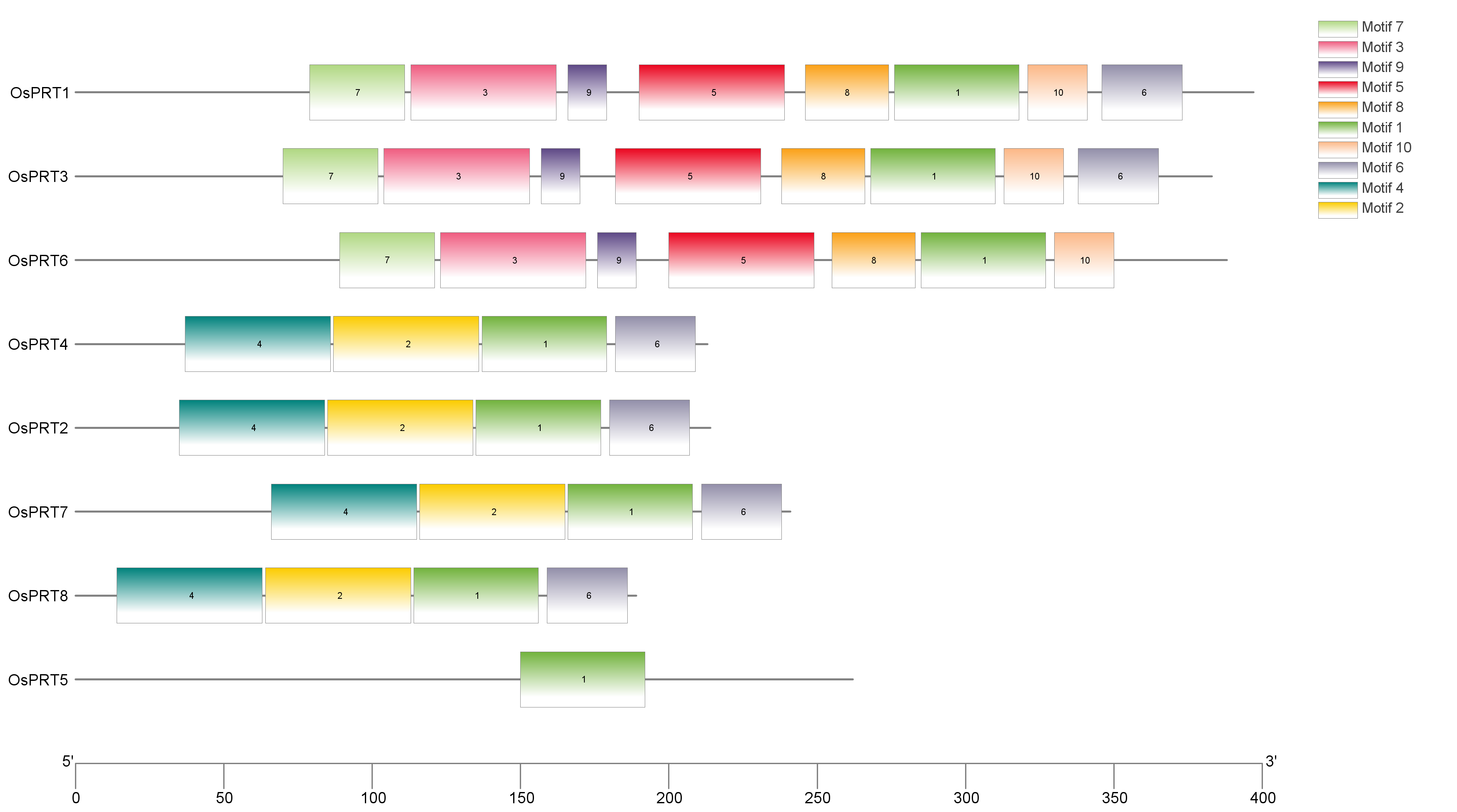


Figure.S1 Distribution of Non-synonymous Mutations in Conserved Motifs

Supplement: Supplementary file 2 — Supplementary Material 2. [file 12870_2026_9070_MOESM2_ESM.docx]
